# Publisher Correction: An Artificial Intelligence-guided signature reveals the shared host immune response in MIS-C and Kawasaki disease

**DOI:** 10.1038/s41467-022-32479-7

**Published:** 2022-08-11

**Authors:** Pradipta Ghosh, Gajanan D. Katkar, Chisato Shimizu, Jihoon Kim, Soni Khandelwal, Adriana H. Tremoulet, John T. Kanegaye, Pediatric Emergency Medicine Kawasaki Disease Research Group, Naomi Abe, Lukas Austin-Page, Amy Bryl, J. Joelle Donofrio-Ödmann, Atim Ekpenyong, Michael Gardiner, David J. Gutglass, Margaret B. Nguyen, Kristy Schwartz, Stacey Ulrich, Tatyana Vayngortin, Elise Zimmerman, Joseph Bocchini, Soumita Das, Jane C. Burns, Debashis Sahoo

**Affiliations:** 1 Department of Cellular and Molecular Medicine, University of California San Diego, San Diego, USA; 2 Department of Medicine, University of California San Diego, San Diego, USA; 3 Department of Pediatrics, University of California San Diego, San Diego, USA; 4 Rady Children’s Hospital-San Diego, San Diego, CA USA; 5 Department of Biomedical informatics, University of California San Diego, San Diego, USA; 6 Department of Computer Science and Engineering, Jacob’s School of Engineering, University of California San Diego, San Diego, USA; 7 Willis-Knighton Health System, Shreveport, LA USA; 8 Department of Pathology, University of California, San Diego, USA; 9 Rady Children’s Hospital San Diego, San Diego, CA USA; 10 Department of Pediatrics, University of California San Diego, San Diego, USA

**Keywords:** Systems analysis, Infectious diseases

Correction to: *Nature Communications* 10.1038/s41467-022-30357-w, published online 16 May 2022

The original version of this Article omitted from the author list the 9th, 10th, 11th and 12th authors Joseph Bocchini (Willis-Knighton Health System, Shreveport, LA), Soumita Das (Department of Pathology, University of California San Diego), Jane C. Burns (Department of Pediatrics, University of California San Diego and the Rady Children’s Hospital-San Diego, San Diego, CA) and Debashis Sahoo (Department of Pediatrics, University of California San Diego and the Department of Computer Science and Engineering, Jacob’s School of Engineering, University of California San Diego). Additionally, the original version of this Article omitted to indicate Jane C. Burns and Debashis Sahoo as co-corresponding authors together with Pradipta Ghosh. The contact information for the corresponding authors of this Article is ‘Jane C. Burns, M.D.; Professor, Department of Pediatrics, Director, Kawasaki Disease Research Center, University of California San Diego; 9500 Gilman Dr. MC 0641, La Jolla, CA 92093-0641 Phone: 858-246-0155: Email: jcburns@health.ucsd.edu’, ‘Debashis Sahoo, Ph.D; Associate Professor, Department of Pediatrics, University of California San Diego; 9500 Gilman Drive, MC 0703, Leichtag Building 132; La Jolla, CA 92093-0703 Phone: 858-246-1803: Fax: 858-246-0019: Email: dsahoo@ucsd.edu’ and ‘Pradipta Ghosh, M.D.; Professor, Departments of Medicine, and Cell and Molecular Medicine, University of California San Diego; 9500 Gilman Drive (MC 0651), George E. Palade Bldg, Rm 232, 239; La Jolla, CA 92093. Phone: 858-822-7633: Fax: 858-822-7636: Email: prghosh@ucsd.edu’. Furthermore, the list of members of the Pediatric Emergency Medicine Kawasaki Disease Research.

Group provided at the end of the Article erroneously included Joseph Bocchini, Soumita Das, Jane C. Burns and Debashis Sahoo.

Finally, the Acknowledgements section erroneously reported the grants iDASH U54HL108460 and R01HL140898 being awarded to J.C.B. and A.H.T. The correct grants awarded to J.C.B and A.H.T. are PreVAIL R61HD105590 and R01HL140898.

These errors have been corrected in both the PDF and HTML versions of the Article.

