# Supplementary material for: An Artificial Intelligence-guided signature reveals the shared host immune response in MIS-C and Kawasaki disease
Source: Nat Commun. 2022 May 16;13:2687. doi: 10.1038/s41467-022-30357-w (PMC9110726; doi:10.1038/s41467-022-30357-w)
Supplement: Supplementary file 1 — Supplementary information [file 41467_2022_30357_MOESM1_ESM.pdf]

SUPPLEMENTARY INFORMATION

An Artificial Intelligence-guided signature reveals the shared host immune response in MIS-C and Kawasaki disease

Authors: Pradipta Ghosh[1,2*¶], Gajanan D. Katkar[1¶], Chisato Shimizu[3,4 ¶], Jihoon Kim[5], Soni Khandelwal[6], Adriana H. Tremoulet[3,4], John T. Kanegaye[3,4], Pediatric Emergency Medicine Kawasaki Disease Research Group[†], Joseph Bocchini[7], Soumita Das[8], and Jane C. Burns[3,4*], Debashis Sahoo[3,6*]

Affiliations:

[1]Department of Cellular and Molecular Medicine, University of California San Diego.
[2]Department of Medicine, University of California San Diego.
[3]Department of Pediatrics, University of California San Diego.
[4]Rady Children's Hospital-San Diego, San Diego, CA
[5]Department of Biomedical informatics, University of California San Diego.
[6]Department of Computer Science and Engineering, Jacob's School of Engineering, University of California San Diego.
[7]Willis-Knighton Health System, Shreveport, LA
[8]Department of Pathology, University of California San Diego.
[†]A list of authors and their affiliations appears at the end of the paper.

[¶] Equal contribution

*Correspondence to:

Pradipta Ghosh, M.D.; Professor, Departments of Medicine, and Cell and Molecular Medicine, University of California San Diego; 9500 Gilman Drive (MC 0651), George E. Palade Bldg, Rm 232, 239; La Jolla, CA 92093. Phone: 858-822-7633: Fax: 858-822-7636: Email: prghosh@ucsd.edu

Debashis Sahoo, Ph.D; Associate Professor, Department of Pediatrics, University of California San Diego; 9500 Gilman Drive, MC 0703, Leichtag Building 132; La Jolla, CA 92093-0831.
Phone: 858-246-1803: Fax: 858-246-0019: Email: dsahoo@ucsd.edu

Jane C. Burns, M.D.; Professor, Department of Pediatrics, Director, Kawasaki Disease Research Center**,** University of California San Diego; 9500 Gilman Dr. MC 0641, La Jolla, CA 92093-0641
Phone: 858-246-0155: Email: jcburns@health.ucsd.edu

CATALOG OF SUPPLEMENTARY MATERIALS

1. *Supplementary Figures and Legends (S1-S4) (Page 3-6)*
2. *Supplementary References (Page 7)*

Supplementary Figures

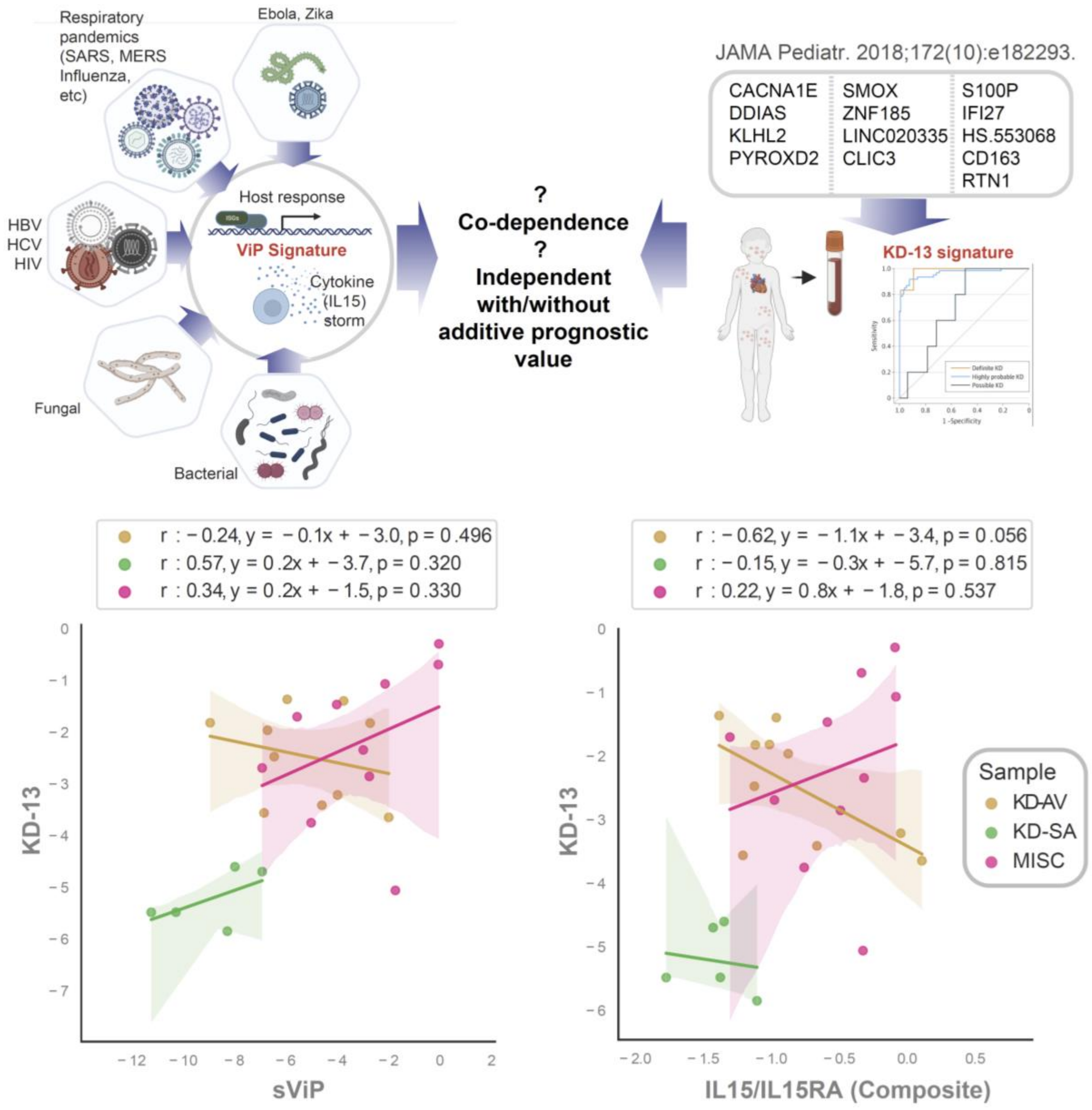

Supplementary Figure S1 (related to Figure 2).

The KD-specific (13 gene)[1] and the sViP (20 gene) signatures are independent. *Top*: Both sViP and KD-13 signatures are induced in the whole blood samples from MIS-C and KD subjects (**Figure 2c, h**), and here we asked if their induction may be co-dependent or independent using a correlation coefficient analysis. *Bottom*: Correlation tests (two-sided test of the slope of the regression line compared to zero) between KD-13 signature (Y axis) and sViP (left, x axis) or IL15/IL15RA compositive score (right, x axis) were calculated and displayed as scatter plots using python seaborn lmplots. The confidence interval around the regression line is indicated with shades.

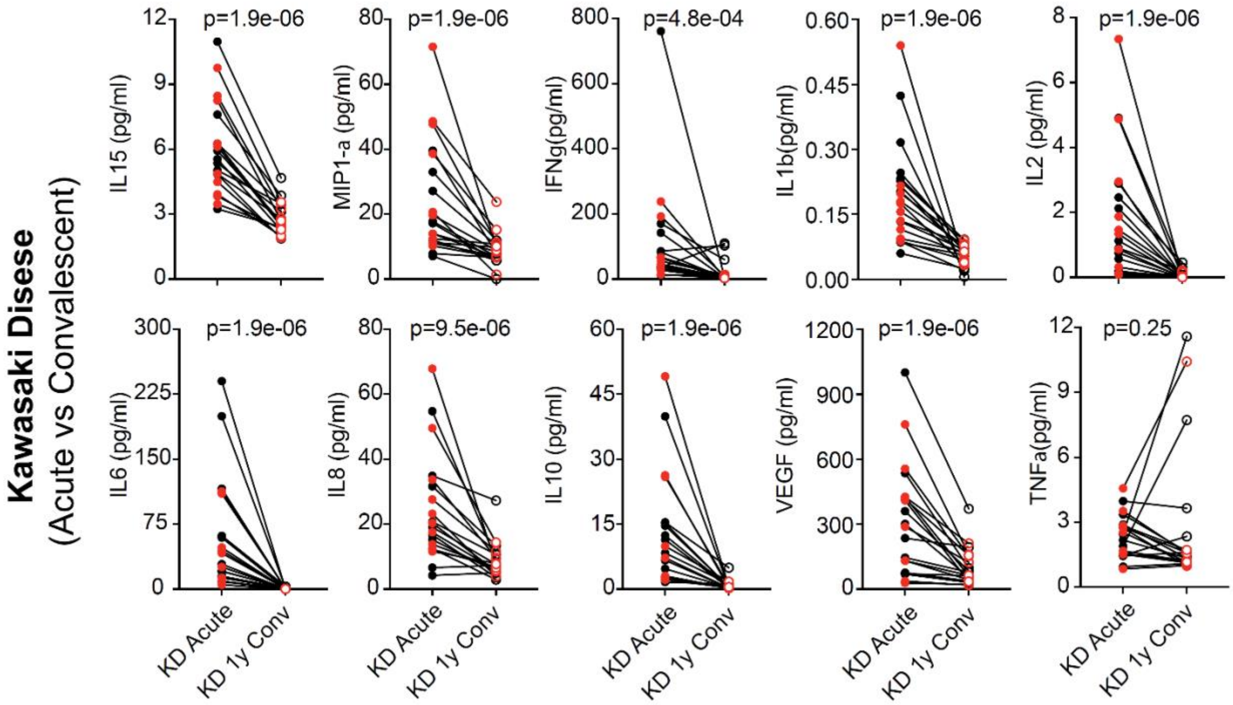

Supplementary Figure S2 (related to Figure 4a).

Serum cytokine profiles in Kawasaki Disease (KD) in acute and convalescent disease. Line plots show the changes in the levels of cytokines in paired samples from acute and convalescent visits of KD subjects. Wilcoxon matched pairs signed rank test was used for two parameter two-sided statistical analysis to test significance. Source data are provided as **Supplementary Data 2.**

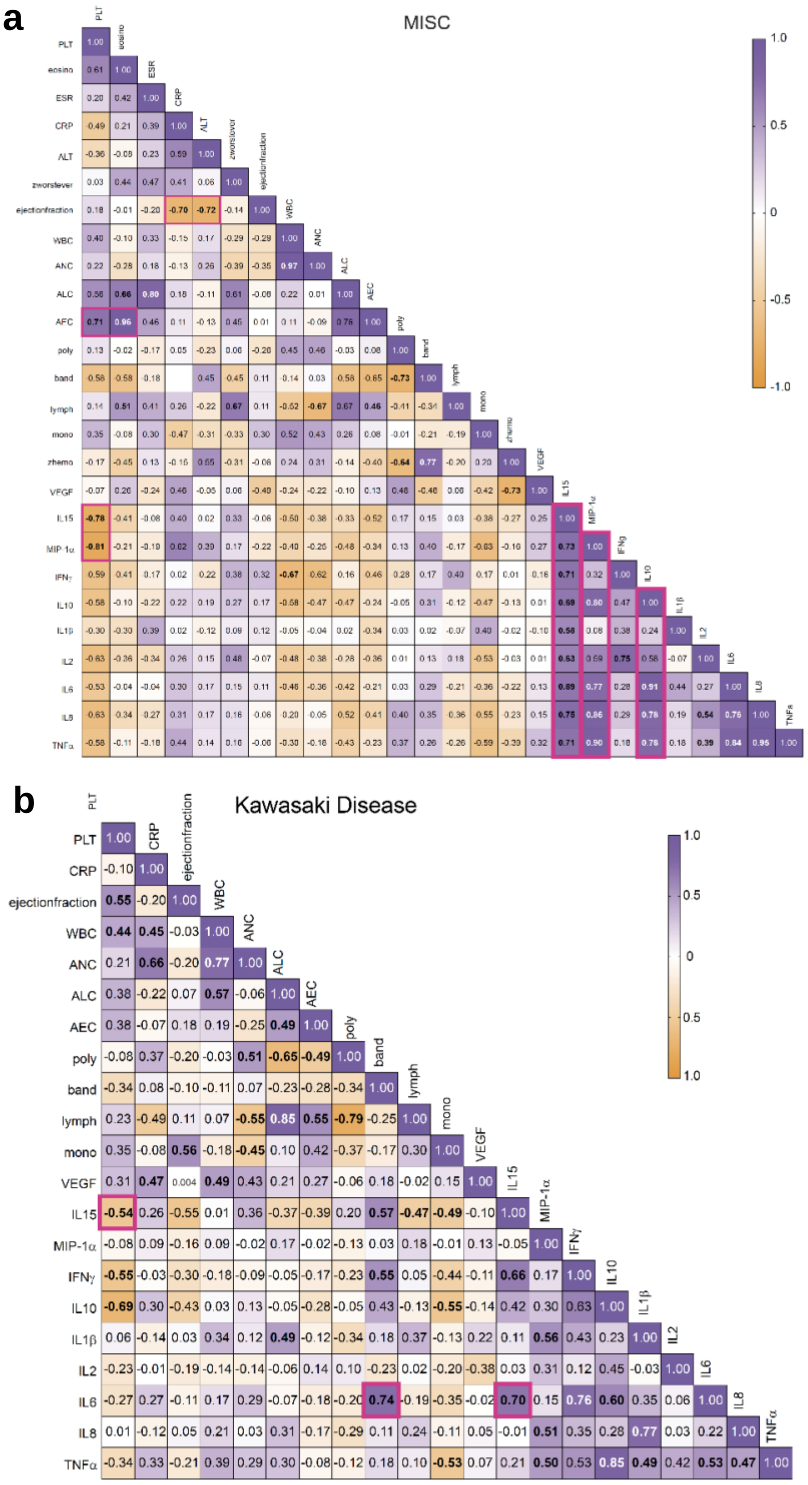

Supplementary Figure S3 (related to Figure 5).
Correlation tests of cytokine levels, as determined by mesoscale and clinical/laboratory findings. Heatmaps of correlation matrix are displayed for MISC (a) and KD (b). Correlation matrix and significance was determined using GraphPad Prism 9. Significant correlations (based on two-sided test of the slope of the regression line compared to zero), defined as those in which p values are < 0.05 are highlighted in bold fonts. Source data are provided as **Supplementary Data 2**.

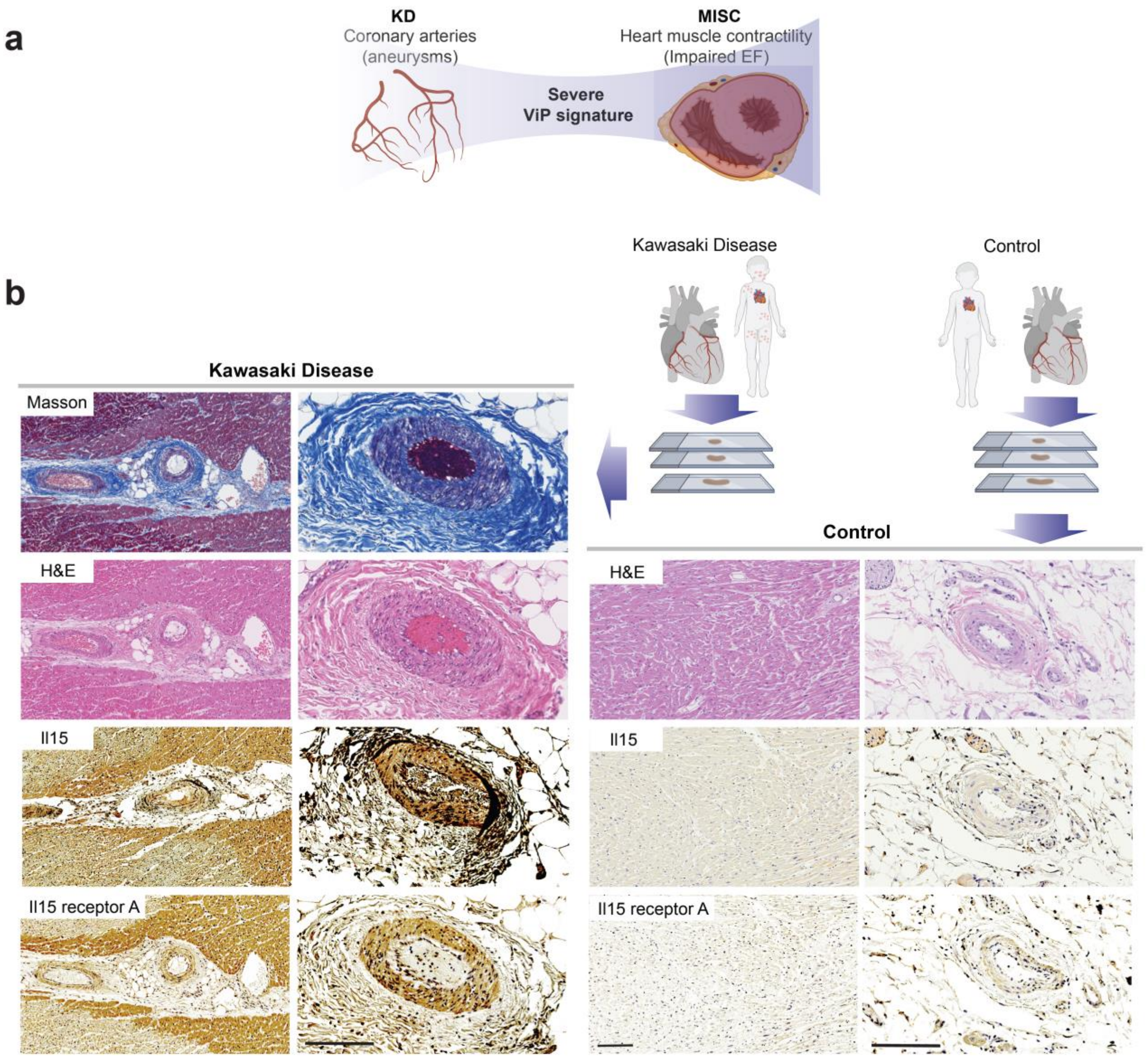

Supplementary Figure S4 (related to Figure 6).

a. Schematic summarizes how sViP signature classifies two distinct cardiac phenotypes, CAA and reduced pump function in KD and MIS-C, respectively. **b**. Consecutive sections from formalin fixed paraffin embedded (FFPE) cardiac tissue collected at autopsy from a child with severe debilitating cardiac complications of KD were stained for masson's trichrome, H&E and IL15/IL15 receptor A antigens (left). Healthy heart sections from another child were used as controls (right). Representative images from one subject is shown. Scale bar = 200 µm.

Supplementary References

1. Wright, V.J. et al. Diagnosis of Kawasaki Disease Using a Minimal Whole-Blood Gene Expression Signature. *JAMA Pediatr* **172**, e182293 (2018).
