# Supplementary material for: An Artificial Intelligence-guided signature reveals the shared host immune response in MIS-C and Kawasaki disease
Source: Nat Commun. 2022 May 16;13:2687. doi: 10.1038/s41467-022-30357-w (PMC9110726; doi:10.1038/s41467-022-30357-w)
Supplement: Supplementary file 3 — Description of Additional Supplementary file [file 41467_2022_30357_MOESM3_ESM.docx]

# An Artificial Intelligence-guided signature reveals the shared host immune response in MIS-C and Kawasaki disease

**Authors:** Pradipta Ghosh^1,2^*^¶^, Gajanan D. Katkar^1¶^, Chisato Shimizu^3,4 ¶^, Jihoon Kim^5^, Soni Khandelwal^6^, Adriana H. Tremoulet^3,4^, John T. Kanegaye^3,4^, Pediatric Emergency Medicine Kawasaki Disease Research Group^7^, Joseph Bocchini^8^, Soumita Das^9^, and Jane C. Burns^3,4^*, Debashis Sahoo^3,6^*

**Affiliations:**

^1^Department of Cellular and Molecular Medicine, University of California San Diego.

^2^Department of Medicine, University of California San Diego.

^3^Department of Pediatrics, University of California San Diego.

^4^Rady Children's Hospital-San Diego, San Diego, CA

^5^Department of Biomedical informatics, University of California San Diego.

^6^Department of Computer Science and Engineering, Jacob’s School of Engineering, University of California San Diego.

^7^A list of authors and their affiliations appears at the end of the paper.

^8^Willis-Knighton Health System, Shreveport, LA

^9^Department of Pathology, University of California San Diego.

^¶^ Equal contribution

***Correspondence to**:

**Pradipta Ghosh, M.D.;** Professor, Departments of Medicine, and Cell and Molecular Medicine, University of California San Diego; 9500 Gilman Drive (MC 0651), George E. Palade Bldg, Rm 232, 239; La Jolla, CA 92093. Phone: 858-822-7633: Fax: 858-822-7636: Email: [prghosh@ucsd.edu](https://hsmail.ucsd.edu/owa/redir.aspx?C=TdXORuJOJYsSaKSg42oiljeLcKpf8UvqUqysJDu31kkqChk7oErWCA..&URL=mailto%3aprghosh%40ucsd.edu)

**Debashis Sahoo, Ph.D;** Associate Professor, Department of Pediatrics, University of California San Diego; 9500 Gilman Drive, MC 0703, Leichtag Building 132; La Jolla, CA 92093-0831. 
Phone: 858-246-1803: Fax: 858-246-0019: Email: [dsahoo@ucsd.edu](mailto:dsahoo@ucsd.edu)

**Jane C. Burns, M.D.;** Professor, Department of Pediatrics, Director, Kawasaki Disease Research Center**,** University of California San Diego; 9500 Gilman Dr. MC 0641, La Jolla, CA 92093-0641

Phone: 858-246-0155: Email: [jcburns@health.ucsd.edu](mailto:jcburns@health.ucsd.edu)

**Description of Additional Supplementary Files**

File Name: Supplementary Data 1

Description: Characteristics of patients and cohorts.

File Name: Supplementary Data 2

Description: Serum cytokines detected using Meso Scale Discovery Electrochemiluminescence (MSD-ECL) Ultra-Sensitive Biomarker Assay in acute KD and MIS-C patients.

File Name: Supplementary Data 3

Description: List of differential expressed genes in MIS-C and KD.
