# Supplementary material for: An Artificial Intelligence-guided signature reveals the shared host immune response in MIS-C and Kawasaki disease
Source: Nat Commun. 2022 May 16;13:2687. doi: 10.1038/s41467-022-30357-w (PMC9110726; doi:10.1038/s41467-022-30357-w)
Supplement: Supplementary file 7 — Reporting Summary [file 41467_2022_30357_MOESM7_ESM.pdf]

nature portfolio

Corresponding author(s): Debashis Sahoo, Jane C Burns, Pradipta Ghosh

Last updated by author(s): 2022/04/05

Reporting Summary

Nature Portfolio wishes to improve the reproducibility of the work that we publish. This form provides structure for consistency and transparency in reporting. For further information on Nature Portfolio policies, see our Editorial Policies and the Editorial Policy Checklist.

Statistics

For all statistical analyses, confirm that the following items are present in the figure legend, table legend, main text, or Methods section.

n/a	Confirmed	
☐	☒	The exact sample size (*n*) for each experimental group/condition, given as a discrete number and unit of measurement
☐	☒	A statement on whether measurements were taken from distinct samples or whether the same sample was measured repeatedly
☐	☒	The statistical test(s) used AND whether they are one- or two-sided<br>*Only common tests should be described solely by name; describe more complex techniques in the Methods section.*
☐	☒	A description of all covariates tested
☐	☒	A description of any assumptions or corrections, such as tests of normality and adjustment for multiple comparisons
☐	☒	A full description of the statistical parameters including central tendency (e.g. means) or other basic estimates (e.g. regression coefficient) AND variation (e.g. standard deviation) or associated estimates of uncertainty (e.g. confidence intervals)
☐	☒	For null hypothesis testing, the test statistic (e.g. *F*, *t*, *r*) with confidence intervals, effect sizes, degrees of freedom and *P* value noted<br>*Give P values as exact values whenever suitable.*
☒	☐	For Bayesian analysis, information on the choice of priors and Markov chain Monte Carlo settings
☒	☐	For hierarchical and complex designs, identification of the appropriate level for tests and full reporting of outcomes
☒	☐	Estimates of effect sizes (e.g. Cohen's *d*, Pearson's *r*), indicating how they were calculated

Our web collection on statistics for biologists contains articles on many of the points above.

Software and code

Policy information about availability of computer code

Data collection	All datasets are available in Gene Expression Omnibus. New Dataset was deposited in GSE178491.
Data analysis	Instructions for how to analyze dataset is available at https://github.com/sahoo00/BoNE

For manuscripts utilizing custom algorithms or software that are central to the research but not yet described in published literature, software must be made available to editors and reviewers. We strongly encourage code deposition in a community repository (e.g. GitHub). See the Nature Portfolio guidelines for submitting code & software for further information.

Data

Policy information about availability of data

All manuscripts must include a data availability statement. This statement should provide the following information, where applicable:

- Accession codes, unique identifiers, or web links for publicly available datasets
- A description of any restrictions on data availability
- For clinical datasets or third party data, please ensure that the statement adheres to our policy

All datasets are available in Gene Expression Omnibus. New Dataset was deposited in GSE178491.

Field-specific reporting

Life sciences study design

All studies must disclose on these points even when the disclosure is negative.

Sample size	All available samples were used.
Data exclusions	No data were excluded from the analysis.
Replication	At least three biological replicates were used in each experiments.
Randomization	Sample allocation were controlled based on disease subtype. Healthy, KD, MISC samples were used.
Blinding	Blinding was performed between two groups: Burns and Sahoo/Ghosh. Burns group knew the disease status of each samples. Sahoo/Ghosh group analyzed samples without prior knowledge of disease status of each samples.

Reporting for specific materials, systems and methods

We require information from authors about some types of materials, experimental systems and methods used in many studies. Here, indicate whether each material, system or method listed is relevant to your study. If you are not sure if a list item applies to your research, read the appropriate section before selecting a response.

Materials & experimental systems

n/a	Involved in the study	
☐	☒	Antibodies
☒	☐	Eukaryotic cell lines
☒	☐	Palaeontology and archaeology
☒	☐	Animals and other organisms
☐	☒	Human research participants
☒	☐	Clinical data
☒	☐	Dual use research of concern

Methods

n/a	Involved in the study	
☒	☐	ChIP-seq
☒	☐	Flow cytometry
☒	☐	MRI-based neuroimaging

Antibodies

Antibodies used	anti-human IL15RA polyclonal antibody (11:200 dilution; proteintech®, Rosemont, IL, USA; catalog# 16744-1-AP)<br>anti-human IL15 monoclonal antibody (1:10 dilution; Santa Cruz Biotechnology, Inc., Dallas, TX, USA; catalog# sc-8437)
Validation	Both antibodies anti-human IL15 and anti-human IL15RA are validated by the suppliers for IHC.

Human research participants

Policy information about studies involving human research participants

Population characteristics	KD: All KD subjects met the American Heart Association (AHA) criteria for complete or incomplete KD.<br>MIS-C: All MIS-C patients met the case definition from the Centers for Disease Control and Prevention.<br>Febrile control patients had fever of at least three days duration and at least one mucocutaneous feature of KD including rash, conjunctival injection, or mucosal erythema. Supplementary Data 1 contains sex, number and age of each cohorts. Patients were not compensated for their participation in this study.
Recruitment	KD and MIS-C Subjects were enrolled prospectively with collection of acute, pre-treatment samples.<br>All febrile controls were enrolled prior to the onset of the pandemic.
Ethics oversight	The study protocol was reviewed and approved by the institutional review board at UCSD (UCSD # 14020). Written informed consent from the parents or legal guardians and assent from patients were obtained as appropriate.

Note that full information on the approval of the study protocol must also be provided in the manuscript.
